# Anti-plant Defense Response Strategies Mediated by the Secondary Symbiont *Hamiltonella defensa* in the Wheat Aphid *Sitobion miscanthi*

**DOI:** 10.3389/fmicb.2019.02419

**Published:** 2019-10-25

**Authors:** Qian Li, Jia Fan, JingXuan Sun, Yong Zhang, MaoLin Hou, JuLian Chen

**Affiliations:** ^1^State Key Laboratory for Biology of Plant Diseases and Insect Pests, Institute of Plant Protection, Chinese Academy of Agricultural Sciences, Beijing, China; ^2^MARA-CABI Joint Laboratory for Bio-Safety, Institute of Plant Protection, Chinese Academy of Agricultural Sciences, Beijing, China

**Keywords:** *Hamiltonella defensa*, *Sitobion miscanthi*, anti-plant defense, defense pathway, enzyme

## Abstract

Bacterial symbionts are omnipresent in insects, particularly aphids, and often exert important effects on the host ecology; however, examples of symbionts that mediate herbivore-plant interactions remain limited. Here, three clones with identical genetic backgrounds were established: a *Hamiltonella defensa*-free clone, *H. defensa-*infected clone and *H. defensa*-cured clone. *H. defensa* infection was found to increase the fitness of *Sitobion miscanthi* by increasing the total number of offspring and decreasing the age of first reproduction. Furthermore, gene expression studies and phytohormone measurement showed that feeding by the *Hamiltonella*-infected clone suppressed the salicylic acid (SA)- and jasmonic acid (JA)-related defense pathways and SA/JA accumulation in wheat plants relative to feeding by the other two clones. Additionally, after feeding by the *Hamiltonella*-infected clone, the activity levels of the defense-related enzymes polyphenol oxidase (PPO) and peroxidase (POD) in wheat plants were significantly decreased compared with the levels observed after feeding by the other two clones. Taken together, these data reveal for the first time the potential role of *H. defensa* of *S. miscanthi* in mediating the anti-plant defense responses of aphids.

## Introduction

Virtually all plants in nature protect themselves against a variety of insect species by using different strategies, including constitutive defense and induced defense mechanisms (Hall, 1999). Constitutive defenses, which are effective against generalist herbivores, are based on secondary metabolites of diverse chemical origins (Schoonhoven et al., 2005). In addition to constitutive defenses, which are always present in the plant, the induction of defenses by herbivorous insect attack is mediated mainly by phytohormones such as jasmonic acid (JA) and salicylic acid (SA). The current theory posits that the JA pathway is frequently induced by chewing-biting herbivores and necrotrophic pathogens (Kawazu et al., 2012) and that the SA pathway is primarily induced by piercing-sucking herbivores and biotrophic pathogens (Glazebrook, 2005).

As one of the largest groups of phloem-feeding insects, aphids (Hemiptera: Aphidoidea) are economically important pests that cause enormous agricultural losses worldwide. The induction of SA-related defense pathways by aphid feeding has been demonstrated in many aphid-plant interactions, for example, for the green peach aphid (*Myzus persicae*) in *Arabidopsis* (Moran and Thompson, 2001), the greenbug aphid (*Schizaphis graminum*) in sorghum (Zhu-Salzman et al., 2004) and the potato aphid (*Macrosiphum euphorbiae*) in tomato (Martinez et al., 2003). Moreover, several genes, such as lipoxygenase (LOX), involved in the JA-related defense pathway are induced by the feeding of the cabbage aphid (*Brevicoryne brassicae*) on wild cabbage (Li et al., 2016) and the feeding of the soybean aphid (*Aphis glycines*) on soybean (Selig et al., 2016).

As a result of the long-term coevolution of plants and herbivores, some herbivores have evolved innovative methods of avoiding detection or manipulating plant defenses (Walling, 2010). This evolution has been demonstrated by findings indicating that some herbivores produce effector molecules to suppress plant defenses in response to phloem feeding (Zhang et al., 2013), oral secretions (Consales et al., 2012; Chung et al., 2013) and egg deposition (Bruessow et al., 2010). Recently, insect-associated symbionts, which usually play hidden roles in insect-plant interactions (Frago et al., 2012), have been recognized as potential agents through which insects mediate plant defense responses (Oliver et al., 2014).

Many insects harbor various types of maternally inherited microbial symbionts (Oliver et al., 2014) that provide essential nutrients and/or have important effects on host insect ecology and physiology (Chen et al., 2000; Montllor et al., 2002; Russell and Moran, 2005; Sakurai et al., 2005; Oliver et al., 2010, 2014; Guidolin and Cônsoli, 2017). Additionally, emerging evidence has revealed that insect-associated symbionts may suppress plant defense responses (Barr et al., 2010; Su et al., 2016) and detoxify plant secondary metabolites and even chemical pesticides (Oliver et al., 2010; Berasategui et al., 2016; Cheng et al., 2017). Therefore, these symbionts may have potential functions in manipulating the “arms race” between insects and host plants. The strategies through which herbivore-associated microbial symbionts manipulate the antagonistic crosstalk between insects and plant defense responses require greater attention.

The grain aphid, *Sitobion miscanthi* Takahashi, is one of the most widespread wheat aphids in China and frequently harbors several secondary symbionts (S-symbionts). *Hamiltonella defensa*, a well-studied S-symbiont in *Acyrthosiphon pisum* that confers conditional adaptive advantages to its host by protecting the insect host against natural enemies (Oliver et al., 2003, 2005; Ferrari et al., 2004), was also detected in *S. miscanthi* in China (Li et al., 2014). Sporadic reports have noted that insect symbionts mediate plant defense responses (Barr et al., 2010; Chung et al., 2013; Su et al., 2016), but there is still relatively little information available on a clear role for *H. defensa* in regulating aphid-plant interactions, especially in the wheat aphid *S. miscanthi*.

In our previous study, *Hamiltonella*-free and *Hamiltonella*-infected aphid clones were established by microinjection, but these clones also carried co-infections with two additional secondary symbionts, *Regella insecticola* and *Spiroplasma* (Li et al., 2018). To rule out the effects of other symbionts on follow-up experiments, an additional antibiotic treatment was performed to construct an aphid clone without S-symbionts other than *H. defensa*. Subsequently, natural *Hamiltonella*-free, *Hamiltonella*-infected and *Hamiltonella*-cured aphid clones without any other S-symbionts were also established through an improved antibiotic treatment. To identify the distribution of *H. defensa* in aphids, we examined the position of *H. defensa* and *Buchnera aphidicola* in the embryo of *S. miscanthi* using fluorescence *in situ* hybridization (FISH). Ecological fitness indices were compared among the *Hamiltonella*-free, *Hamiltonella*-infected and *Hamiltonella*-cured clones. Finally, the effects of *H. defensa* on the expression of JA- and SA-related defense pathway genes, SA and JA accumulation and the activity of defense-related enzymes in plants were investigated. Our results show that *H. defensa* mediated the anti-plant defense responses by suppressing the expression of SA and JA-related defense pathway genes and SA and JA production in plants and decreasing defense-related enzyme activity in wheat plants, all of which resulted in improved aphid fitness.

## Materials and Methods

### Experimental Aphid Clones

The following clones of *S. miscanthi* established by a single female were used in this study: a *Hamiltonella*-free clone (DZ) was collected from a Dezhou wheat field in Shandong Province, China, and a natural *Hamiltonella*-infected clone (YX) was collected from a Yuxi wheat field in Yunnan Province, China, according to our previous studies (Li et al., 2018).

### *H. defensa* Artificial Infection and Antibiotic Elimination

The injection of *H. defensa* and antibiotic elimination was performed according to our previous work (Li et al., 2018). Then, DZ and DZ-HT contained no known secondary symbionts and DZ-H contained only *H. defensa* were established after an additional antibiotic treatment experiment (Supplementary Method “Details About Microinjection and Antibiotic Treatment”). The infected and cured clones were not used in any experiment until at least 10 generations had passed to eliminate any negative effects associated with mechanical damage and the antibiotic treatment (Koga et al., 2003), and aphids from the infected and cured clones were retested by PCR before the next step in the experiment assay.

### Gene Amplification, Sequencing, and Phylogenetic Analysis

To ensure that the newly infected and cured aphid clones were produced by microinjection and antibiotic treatment rather than contamination and to verify whether the aphid genotype of the new *H. defensa*-acquired *S. miscanthi* DZ-H clone was contaminated, the mitochondrial cytochrome oxidase I (*COI*) sequences of the *S. miscanthi* native *Hamiltonella-*infected YX clone, *Hamiltonella*-free DZ clone, artificial *Hamiltonella-*infected DZ-H clone and *Hamiltonella*-cured DZ-HT clone were amplified and used to build a maximum likelihood tree with Kimura’s two-parameter distance and 1000 bootstrap resampling iterations with MEGA software, version 7.0.26 (Kumar et al., 2001). The nucleotide sequence accession numbers of the *COI* genes from the *S. miscanthi* YX, DZ, DZ-H, and DZ-HT clones were MH805861, MH805862, MH805863, and MH805864, respectively.

To verify the consistent identity of the *H. defensa* strain during artificial infection, we adapted the multilocus approach developed in a previous study (Henry et al., 2013). Two *H. defensa* housekeeping genes (*dnaA* and *recJ*) and one APSE (a bacteriophage produced by *H. defensa*) locus, *P3*, were selected and analyzed in the YX and DZ-H clones. A phylogenetic tree was constructed by using the maximum likelihood method in MEGA. The nucleotide sequences of the *dnaA*, *recJ*, and *P3* genes from the *H. defensa* donor *S. miscanthi* YX clone described in this paper have been deposited in GenBank under the accession numbers MH884762, MH823746, and MH910617. The primer information is listed in Supplementary Table S1. The cycling conditions were 94°C for 4 min; followed by 35 cycles of 94°C for 30 s, 60°C for 45 s, and 72°C for 1 min; and 4°C for the final elongation. The reaction products were analyzed with a model 3500 ABI PRISM DNA sequencer (Perkin-Elmer, New York, NY, United States).

### Relative Changes in *B. aphidicola* Abundance by Quantitative PCR

After the clones were reared for 10 generations, DNA was extracted from 30 mixed first-instar nymphs of the DZ, DZ-H, and DZ-HT clones, and the *B. aphidicola* relative abundance was quantified by real-time PCR. All data were compared with the DZ clone as a control. The primers used in this study are based on our previous work (Li et al., 2018). Three biological replicates were performed.

### Fluorescence *in situ* Hybridization (FISH)

Fluorescence *in situ* hybridization (FISH) was performed according to a previous study (Sakurai et al., 2005) with some modifications [Supplementary Method “Details About Fluorescence *in situ* Hybridization (FISH)”]. The probe ApisBuch-Cy5 (5′-Cy5-CCTCTTTTGGGTAGATCC-3′) targeted the *16S rRNA* of *Buchnera* spp. (Kumar et al., 2001), and the probe ApisHami-Cy3 (5′-Cy3-CCAGAT TCCCAGACTTTACTCA-3′) targeted the *16S rRNA* of *H. defensa*. To confirm the specificity of the detection, a series of control experiments were conducted as previously described (Qian et al., 2018): *Hamiltonella*-free aphid embryos and *Hamiltonella*-cured aphid embryos were probed with the *H. defensa 16S rRNA* probe to confirm the result of *H. defensa* hybridization.

### Fitness Measurements

Thirty nymphs from the DZ, DZ-H, and DZ-HT clones were selected at random and individually placed in petri dishes containing wheat seedlings whose roots were inserted into water in 1.5 ml tubes and kept at 20°C under a long-day (16 h) light cycle. Fitness indices, including total number of offspring and age of first reproduction, were monitored daily until all nymphs had completed their whole life cycle. We measured the offspring of five newly emerged adults collected from each clone and performed 6 replications.

### Aphid Infestation Treatments

At the two-leaf stage, 30 newly molted adults of the DZ, DZ-H, and DZ-HT clones were transferred to the first leaf (the oldest leaf) of wheat, and the movement of the aphids was restricted by a plastic cage (2.7 cm × 2.7 cm × 2.7 cm) clipped onto the leaf to prevent their escape. Empty cages were clipped onto uninfested plants. Each pot contained one wheat plant and was kept in the culture room at 20 ± 1°C with 75% relative humidity and a light: dark photoperiod of 16:8 (L: D) hours. After 30 min, all aphids had begun settling and feeding, and this time was recorded as 0 h. A 100-mg quantity of plant tissue from each clip-caged leaf was harvested after 24, 48, or 72 h of aphid feeding for RNA extraction and assessed for the induction of genes associated with plant defense, as described below. In addition, plant tissues not subjected to aphid feeding were harvested at the different time points as controls. All treatments had three biological replicates.

### Wheat RNA Extraction and Quantitative Real-Time PCR (qRT-PCR)

Wheat leaf tissues were ground in liquid nitrogen. Total RNA was extracted with an RNeasy Plus kit following the manufacturer’s protocol. RNA concentration was evaluated using a NanoDrop 1000 spectrophotometer, and then 1 μg of RNA was used to synthesize first-stand cDNA using the EasyScript One-Step gDNA Removal and cDNA Synthesis SuperMix (TransGen Biotech, Beijing, China) according to the manufacturer’s protocol. Each cDNA sample was generated in triplicate from each biological replicate. The genes *PR-1*, *PAL* and β*-1,3-GA*, which encode pathogenesis-related protein 1, phenylalanine ammonia lyase, and beta-1,3-glucanase, respectively, were selected as SA-marker genes, and the genes *AOS*, *LOX* and *FAD*, which encode allene oxide synthase, lipoxygenase and Ω-3 fatty acid desaturase, respectively, were selected as JA marker genes because they are strongly induced in response to insects and involved in the synthesis of these two plant hormones (Zhang et al., 2013; Züst and Agrawal, 2016). To quantify the *PR-1*, *PAL*, β*-1,3-GA*, *AOS*, *LOX*, and *FAD* transcript levels, qRT-PCR was performed. All treatments had three biological replicates, and each replicate consisted of three technical replicates. The primers for the SA and JA marker genes are shown in Supplementary Table S1. The abundances of the SA and JA marker genes were normalized to that of the plant housekeeping gene β*-actin* to obtain the relative abundances. All data were compared with the control to obtain the relative value.

### Quantification of SA and JA

Wheat leaves were subjected to feeding by *Hamiltonella*-free, *Hamiltonella*-infected and *Hamiltonella*-cured aphids as described above. Ten leaves were used for each treatment, and leaf tissues were harvested 24, 48, and 72 h after placing aphids from three individual plants were pooled as one biological replicate per clone and time point. Three biological replicates were performed. SA and JA were extracted and measured using GC/MS as described previously (Tooker et al., 2008).

### Enzyme Activity Assays

The methods used to determine the wheat plant defense-related enzyme activities were largely the same as previously reported methods (Lang et al., 2017). The activities of POD and PPO were evaluated using their respective diagnostic kits (Nanjing Jiancheng Bioengineering Institute, Nanjing, China). POD activity was determined by a spectrophotometer (UV-2000, UNICO, Shanghai, China) following the change in absorption at 420 nm due to guaiacol oxidation (Maehly and Chance, 1995). The PPO activity was assayed according to the methods of a previous study (Cai et al., 2010). Each test had three biological replicates.

### Statistical Analysis

Treatment effects were assessed with an analysis of variance (ANOVA) or Student’s *t*-test using IBM SPSS statistics version 21 (ver. 21, SPSS, Inc., Chicago, IL, United States). For assays in which two or more treatments were compared, Tukey’s honestly significant difference (HSD) multiple comparison test (*P* < 0.05) was used to determine whether the treatments were significantly different.

## Results

### Establishment of Aphid Clones

**FIGURE 1:**
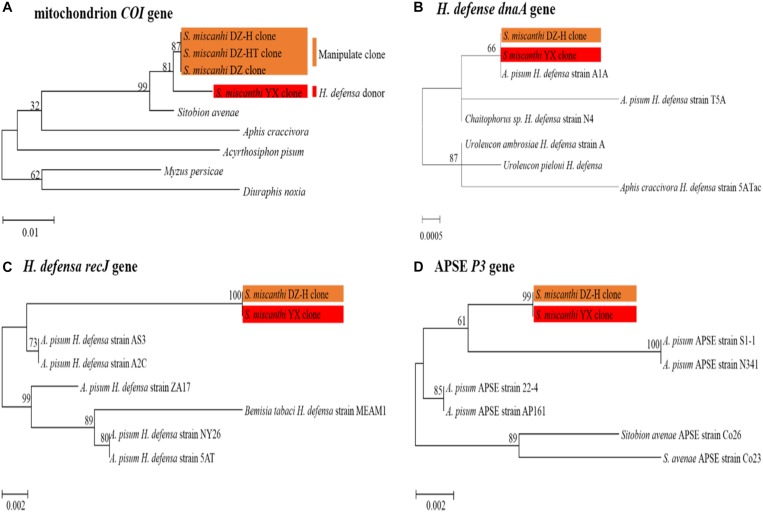
Phylogenetic trees were constructed with molecular evidence. **(A)** Phylogenetic analysis of different clones based on the *COI* gene. **(B–D)** The maximum likelihood (ML) tree was constructed based on *H. defensa* and APSE multilocus gene sequences. The orange frame indicates the manipulated clones, and the red frame indicates the *H. defensa* donor *Sitobion miscanthi* YX clone. The bar indicates the estimated number of substitutions per site.

Our previous work revealed that the YX clone was infected with three S-symbionts—*R. insecticola*, *H. defensa* and *Spiroplasma* sp.—and that the DZ clone was infected with *R. insecticola* and *Spiroplasma* sp. With the improvement of previous manipulation methods, a *Hamiltonella*-infected clone (DZ-H) and a new *Hamiltonella*-cured clone (DZ-HT), which had an identical genetic background to the DZ clone, were constructed. To rule out the effects of other S-symbionts on the following experiments, an additional antibiotic treatment was performed to eliminate S-symbionts other than *H. defensa* in the DZ, DZ-H, and DZ-HT clones. PCR detection revealed that *H. defensa* was completely cured in the DZ-HT clone after antibiotic treatment (Supplementary Figure S1). To rule out contamination of the new *Hamiltonella*-infected DZ-H clone and *Hamiltonella*-cured DZ-HT by the native *Hamiltonella*-infected YX clone and *Hamiltonella*-free DZ clone, the mitochondrial cytochrome oxidase I (*COI*) sequences of the YX, DZ, DZ-H, and DZ-HT clones were aligned with MEGA software, and a maximum likelihood tree was built (Figure 1A). The results showed that the sequence of the *COI* mitochondrial gene was strictly identical among the DZ, DZ-H, and DZ-HT clones but distinct in the YX clone, indicating that the newly infected and cured *H. defensa* aphid clones were derived from microinjection and antibiotic treatment.

To verify the consistent identity of the *H. defensa* strain during microinjection, a phylogenetic tree was constructed by using two *H. defensa* housekeeping genes (*dnaA* and *recJ*) and one APSE (a bacteriophage secreted from *H. defensa*) gene (*P3*). The results showed that all of the orthologous genes were completely identical in the native *Hamiltonella*-infected *S. miscanthi* YX clone and artificial *Hamiltonella*-infected DZ-H clone (Figures 1B–D). These findings indicated that the *H. defensa* strain remained consistent throughout the microinjection process.

### Relative Abundance of *B. aphidicola* in the DZ, DZ-H, and DZ-HT Clones

**FIGURE 2:**
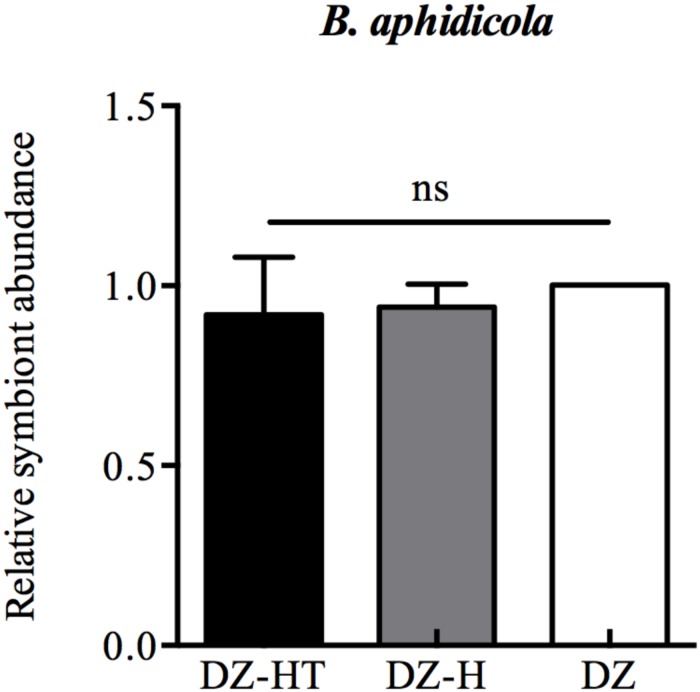
Relative changes in *B. aphidicola* abundance after microinjection and antibiotic treatment designed to specifically infect and eliminate *H. defensa.* qPCR analysis of DNA extracted from microinjection-treated aphids (DZ-H clone), antibiotic-treated aphids (DZ-HT clone), and *Hamiltonella*-free aphids (DZ clone). Microinjection and antibiotic treatments did not have an effect on the abundance of *B. aphidicola*. Bars represent the standard errors of the means, different letters indicate significant differences based on one-way ANOVA followed by Tukey’s HSD multiple comparison test (*P* < 0.01), and ns indicates no significant difference.

Quantitative PCR results showed that at the first instar stage, the relative abundance of *B. aphidicola* in DZ, DZ-H, and DZ-HT were not significantly different after the antibiotic treatment (Figure 2), however, the abundance of *H. defensa* in the DZ-HT clone could not be detected after antibiotic treatment, as was the case in the DZ clone. Moreover, the other S-symbionts were also not detected after an additional antibiotic treatment in all clones (data not shown). These results show that microinjection and a moderate concentration of antibiotics could specifically cure targeted symbionts without affecting other symbionts.

### *In situ* Hybridization of *H. defensa* and *B. aphidicola*

**FIGURE 3:**
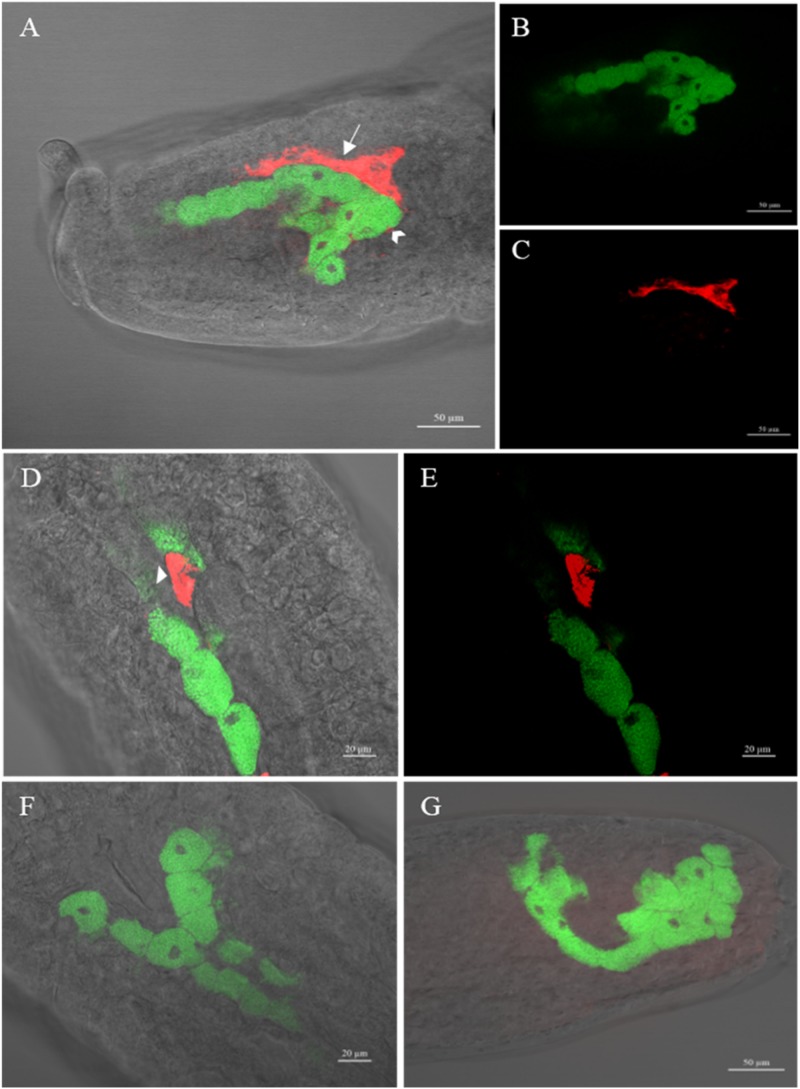
Whole-mount *in situ* hybridization of aphid embryos targeting *H. defensa* (red) and *B. aphidicola* (green). **(A–E)** Different embryos of the artificial *Hamiltonella*-infected aphid clone DZ-H in which sheath cells, secondary bacteriocytes and hemolymph harboring *H. defensa* are seen in addition to a number of bacteriocytes harboring *B. aphidicola*. **(F,G)** An embryo of the *Hamiltonella*-free aphid clone DZ and *Hamiltonella*-cured aphid clone DZ-HT in which *H. defensa* was not detected but *B. aphidicola* was detected. Swallowtail, sheath cell; arrowhead, secondary bacteriocyte; arrow, hemolymph.

Whole-mount FISH revealed the cellular localizations of *H. defensa* and *B. aphidicola* (Figure 3). In aphid embryos of the *Hamiltonella*-infected DZ-H clone, strong signals of the *Hamiltonella*-specific probe (red) were found in sheath cells, secondary mycetocytes and hemolymph (Figures 3A–E). Interestingly, the sheath cells and secondary bacteriocytes were located on the periphery of bacteriocytes, where *B. aphidicola* (green) was harbored. Signals of *B. aphidicola*, but not *H. defensa*, were detected in the *Hamiltonella*-free clone DZ and the *Hamiltonella*-cured clone DZ-HT (Figures 3F,G), and the no-probe control confirmed the specificity of the detected signals (data not shown).

### Fitness Measurements

**FIGURE 4:**
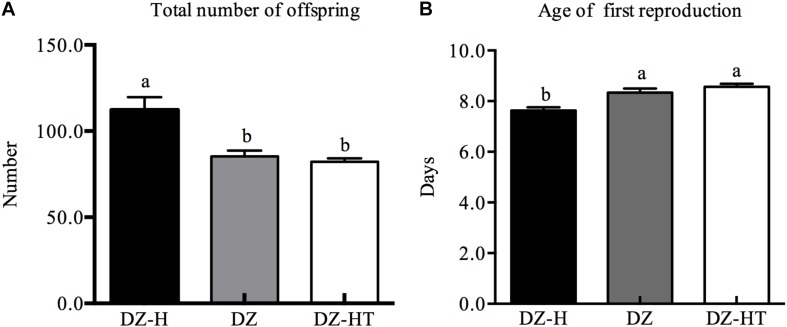
Comparison of aphid fitness measurements among *Hamiltonella*-infected (DZ-H), *Hamiltonella*-free (DZ) and *Hamiltonella*-cured (DZ-HT) *S. miscanthi* clones. **(A)** Total number of offspring. **(B)** Age of first reproduction. Bars represent the standard errors of the means, and different letters above the bars indicate significant differences based on one-way ANOVA followed by Tukey’s HSD multiple comparison test (*P* < 0.05).

Two aphid demographic parameters—the total number of offspring and the age of first reproduction—were compared among the *Hamiltonella*-free clone (DZ), *Hamiltonella*-infected clone (DZ-H) and *Hamiltonella*-cured clone (DZ-HT) with identical genetic backgrounds (Figure 4). The total number of offspring differed significantly among the three clones (*F*_2,15_ = 11.816, *P* < 0.001). With an average number of 112.3 offspring it was significantly higher in the DZ-H clone than in DZ (85.3) and in DZ-HT (82.2) (Figure 4A). Meanwhile, the aphid age of first reproduction also exhibited significant variation among the three clones (*F*_2,87_ = 13.101, *P* < 0.001). With an average age of first reproduction of 7.6 days it was significantly lower in DZ-H clone than in DZ (8.3 days) and DZ-HT (8.6 days) (Figure 4B).

### Effect of *H. defensa* on the Plant SA and JA Defense Pathways

**FIGURE 5:**
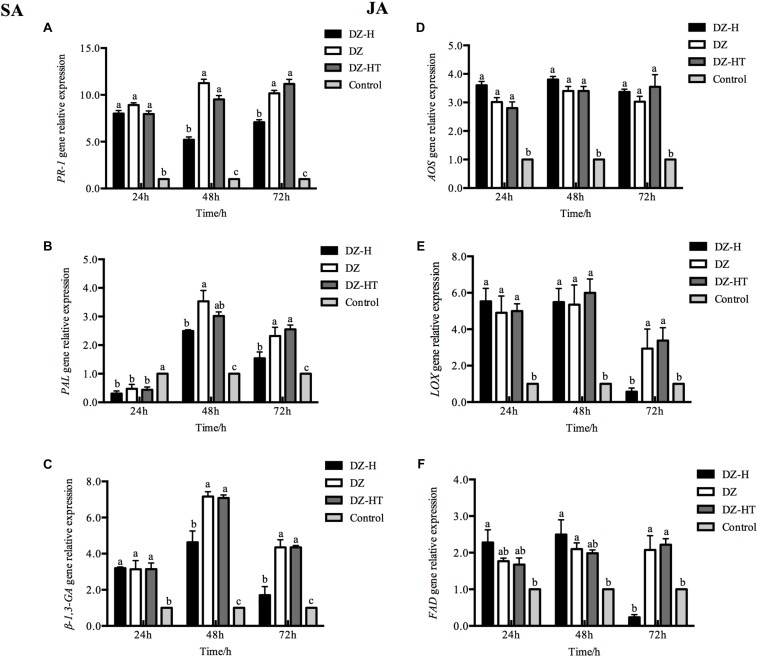
Effect of *H. defensa* infection on the induced plant SA and JA defense pathways. **(A–C)** Expression levels of SA-regulated genes in plants infested by aphids with *H. defensa* infection. **(D–F)** Expression levels of JA-regulated genes in plants infested by aphids with *H. defensa* infection. Bars represent the standard errors of the means, and different letters above the bars indicate significant differences based on one-way ANOVA followed by Tukey’s HSD multiple comparison test (*P* < 0.05).

To investigate whether *H. defensa* suppresses plant defenses, we measured SA/JA defense marker gene expression in wheat plants at different time points after feeding by the DZ, DZ-H, and DZ-HT clones. Some of the key genes involved in the SA and JA defense pathways were found to be differentially expressed in wheat leaves (Figure 5). Compared with the levels observed after feeding by the DZ and DZ-HT clones, the relative levels of expression of the SA-related defense genes *PR-1*, *PAL*, and β*-1,3-GA* in wheat after feeding by the DZ-H clone were not significantly different at 24 h (*PR-1*: *F*_2,6_ = 3.237, *P* = 0.111; *PAL*: *F*_2,6_ = 1.810, *P* = 0.243; β*-1,3-GA*: *F*_2,6_ = 0.026, *P* = 0.975) but were exhibited significant variation at other time points. The results in Figures 5A–C show that the SA-defense-related gene levels were significantly lower in the leaves feeding by DZ-H clone than DZ and DZ-HT at 48 and 72 h (*PR-1*: *F*_2,6_ = 67.541, *P* < 0.001, and *F*_2,6_ = 34.385, *P* = 0.001, for 48 and 72 h, respectively; *PAL*: *F*_2,6_ = 14.608, *P* = 0.005, and *F*_2,6_ = 15.519, *P* = 0.004, for 48 and 72 h, respectively; β*-1,3-GA*: *F*_2,6_ = 37.899, *P* < 0.001, and *F*_2,6_ = 51.814, *P* < 0.001, for 48 and 72 h, respectively).

The relative levels of expression of some JA-related defense genes differed from those of SA-related defense genes. Compared with the levels observed after feeding by the DZ and DZ-HT clones, the relative gene expression levels of *LOX* and *FAD* were not significantly different at 24 and 48 h (*LOX*: *F*_2,6_ = 0.680, *P* = 0.542, and *F*_2,6_ = 0.449, *P* = 0.658, for 24 and 48 h, respectively; *FAD*: *F*_2,6_ = 1.980, *P* = 0.219, and *F*_2,6_ = 1.064, *P* = 0.402, for 24 and 48 h, respectively) but differed significantly at 72 h after feeding by the DZ-H clone. The results in Figures 5D–F show that the JA-defense-related gene levels were significantly lower in the leaves feeding by DZ-H clone than DZ and DZ-HT at 72 h (*LOX*: *F*_2,6_ = 12.174, *P* = 0.008; *FAD*: *F*_2,6_ = 19.762, *P* = 0.002). However, the relative gene expression level of *AOS* in wheat plants was not exhibit significant variation at any of the monitored times after feeding by the DZ, DZ-H, and DZ-HT clones. Both the plant SA- and JA-related defense genes were upregulated after feeding by the DZ, DZ-H, and DZ-HT clones; however, the upregulated expression levels of the relative genes were significantly lower after feeding by the *Hamiltonella*-infected clone than by the *Hamiltonella*-free clone and *Hamiltonella*-cured clone. Therefore, these results indicate that the presence of *H. defensa* in aphids may play a role in suppressing plant defense responses.

### Effect of *H. defensa* on Wheat SA and JA Accumulation

**FIGURE 6:**
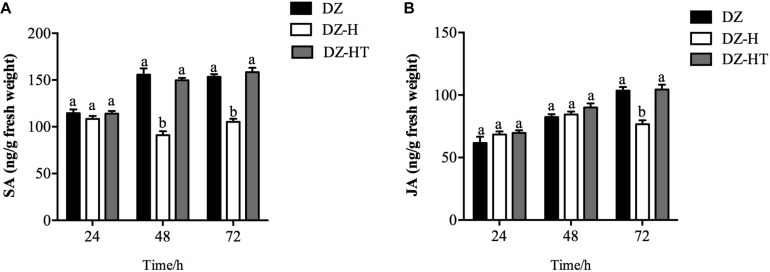
Salicylic acid and JA accumulation in plants infested by aphids with or without *H. defensa* infection. SA level **(A)** and JA level **(B)** were assessed in wheat plants after different periods of infestation with the DZ, DZ-H, and DZ-HT clones. Bars represent standard errors of the means, and different letters above the bars indicate significant differences based on a one-way ANOVA followed by Tukey’s HSD multiple comparison test (*P* < 0.05).

To determine if an antagonistic interaction between the SA and JA signaling pathways is mediated by *H. defensa*, we measured endogenous SA and JA levels in wheat plants at different time points after infestation and feeding by the DZ, DZ-H, and DZ-HT clones. Compared to the DZ and DZ-HT clones, the SA concentrations in wheat subjected to feeding by the DZ-H clone were not significantly different at 24 h (*F*_2,6_ = 1.070, *P* = 0.401) but differed significantly at 48 and 72 h. With an average concentration of 91.1 and 105.3 ng/g it was significantly lower in DZ-H clone than in DZ (155.7 and 153.4 ng/g) and DZ-HT (149.8 and 158.3 ng/g) (Figure 6A) (*F*_2,6_ = 56.653, *P* < 0.001 and *F*_2,6_ = 61.863, *P* < 0.001, for 48 and 72 h, respectively) (Figure 6A). However, the JA concentrations in wheat fed on by the DZ-H clone for 72 h was 105.3 ng/g, which is significantly lower than the wheat after being fed on by clones DZ (153.4 ng/g) or DZ-HT (158.3 ng/g) (*F*_2,6_ = 22.617, *P* = 0.002) (Figure 6B).

### Effect of *H. defensa* on Wheat Defense-Related Enzyme Activity

**FIGURE 7:**
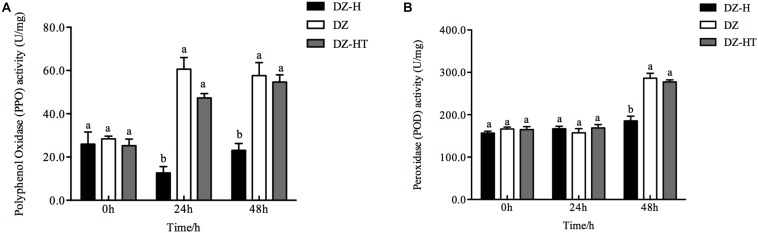
Effect of *H. defensa* on wheat defense-related enzyme activity. Activities of PPO **(A)** and POD **(B)** were assessed in wheat plants after different periods of infestation with the DZ, DZ-H, and DZ-HT clones. Bars represent standard errors of the means, and different letters above the bars indicate significant differences based on a one-way ANOVA followed by Tukey’s HSD multiple comparison test (*P* < 0.05).

To investigate the mechanism by which *H. defensa* improves the fitness of *S. miscanthi* for wheat plants, we measured defense-related enzyme (PPO and POD) activity in plants (Figure 7). After the plants were fed on by the DZ-H clone for 24 and 48 h, the activity of PPO in the plants was 12.7 and 23.1 U/mg, respectively, which were significantly lower than the activity after being fed on by the clones DZ (60.7 and 57.7 U/mg) or DZ-HT (47.3 and 54.7 U/mg) (*F*_2,6_ = 44.723, *P* < 0.001, and *F*_2,6_ = 19.093, *P* = 0.003, for 24 and 48 h, respectively). The activity of POD in plants fed on by the DZ-H clone for 48 h was 185.5 U/mg, which is significantly lower than the activity after being fed on by clones DZ (286.2 U/mg) or DZ-HT (277.5 U/mg) (*F*_2,6_ = 33.394, *P* = 0.001), but there was no significant difference in POD activity among the plants fed on by different clones after 24 h of feeding (*F*_2,6_ = 0.594, *P* = 0.582) (Figures 7A,B).

## Discussion

Insect symbionts are usually hidden players in insect-plant interactions (Frago et al., 2012). Numerous studies have shown that microbial symbionts play important roles in insect survival and confer conditional adaptive advantages, including providing nutrition, protecting the host insect against natural enemies (Oliver et al., 2003, 2005, 2010), and detoxifying toxins (Cheng et al., 2017). In certain insects, symbiotic viruses suppress the host insect defense reaction (Asgari and Johnson, 2000). However, very little is known about how the endosymbionts of herbivores may mediate host plant defenses. Here, we illustrated strategies mediated by aphid symbionts against plant defense responses.

In our previous work, we established a *Hamiltonella*-infected clone and a *Hamiltonella*-reduced clone (Li et al., 2018) and to rule out the effects of remnant *H. defensa* and other S-symbionts, an improved method of antibiotic treatment was performed to completely eliminate *H. defensa* and other S-symbionts without affecting the P-symbiont *B. aphidicola*. In this study, the *Hamiltonella*-infected clone was used, and a new *Hamiltonella*-cured clone that presented a consistent relative abundance of *B. aphidicola* was established. Then, *H. defensa* and *B. aphidicola* were identified in the aphid embryos by FISH. Notably, strong signals of *H. defensa* in the *Hamiltonella*-infected clone DZ-H were found in sheath cells, secondary bacteriocytes and hemolymph, which were located close to the bacteriocytes that contained *B. aphidicola*. This observation in artificial *Hamiltonella*-infected DZ-H clones was similar to our previous study that used the natural *Hamiltonella*-infected YX clone (Qian et al., 2018), indicating the diverse distribution of *H. defensa* in aphids and the lack of connection between infection methods and distribution. The subsequent fitness measurement results showed that infection by *H. defensa* significantly increased the fitness of *S. miscanthi*, as evidenced by the greater total number of offspring and lower age of first reproduction with *H. defensa* infection. This result was consistent with our previous work (Li et al., 2018), and complete elimination of *H. defensa* caused a significant decrease in the total number of offspring, even more so than clones with decreased relative abundance of *H. defensa*. A prior report indicated that a close relationship with symbionts can be costly for the host insect, negatively affecting insect development (Oliver et al., 2006; Vorburger and Gouskov, 2011; Polin et al., 2014). Interestingly, in our study, the fitness of *S. miscanthi* increased after infecting with *H. defensa*. Therefore, we presume that these direct costs may be caused by a trade-off between allocating resources to symbiosis and improving the adaptive ability of the host insect. These results indicate that the change in fitness caused by *H. defensa* infection may be due to the specific strains of *H. defensa* and their host aphid species.

Plants initiate phytohormone biosynthesis and antiherbivore defense responses when attacked by herbivores. Therefore, herbivorous insects have evolved strategies to circumvent plant defense responses; one such strategy is to inhibit the core gene expression of plant defense-related pathways and phytohormone production. Although it has been reported that herbivorous insects overcome plant defenses (Musser et al., 2010), only recently emerging evidence has indicated that the symbionts harbored in host insects mediate plant defense pathways. In maize roots, larvae of the western corn rootworm *Diabrotica virgifera virgifera* influence the downregulation of JA defense pathway genes via infection with *Wolbachia* sp. (Barr et al., 2010). In tomato, larvae of the potato beetle *Leptinotarsa decemlineata* inhibit JA-responsive gene expression and JA accumulation, but this inhibition was not observed when the insects were treated with antibiotics (Chung et al., 2013). Interestingly, a recent study has shown that an indirect suppression response mediated by *H. defensa* occurs in pea aphid through attenuating the yield of host plant volatiles (Frago et al., 2017). Moreover, in whitefly, infection with *H. defensa* resulted in suppression of the plant JA defense response (Su et al., 2016); however, in the present study, we found that the presence of *H. defensa* in *S. miscanthi* significantly decreased the expression of the SA-responsive antiherbivore genes from 48 to 72 h after feeding but suppressed only some JA-responsive genes at 72 h (Figure 5). Meanwhile, SA and JA accumulation also displayed similar tendencies to the results of gene expression measurement. Therefore, in our study, SA is important in establishing resistance early in the infestation process, and JA is important in later facilitation of resistance. It has been well-documented that the two hormones SA and JA are natural antagonists, most likely as part of a plant’s strategy to fine-tune its defenses (Thaler et al., 2012). If one pathway is genetically manipulated, hormonal “crosstalk” can be measured in the other (Züst and Agrawal, 2016). It has been reported that the different hormone signaling pathways are to some extent activated during green peach aphid (*Myzus persicae*) infestation, and the SA signaling pathway is the predominant one (Rodriguez et al., 2014), while JA-responsive genes are repressed (Kerchev et al., 2013). However, the application of JA and SA induction treatments in tomato resulted in increased aphid resistance, suggesting a role for both pathways in the activation of defenses against aphids (Cooper et al., 2004). This result also suggests that the activation of SA signaling by aphid feeding does not necessarily suppress JA-related defenses. It is likely that, in plant-aphid interactions, the JA and SA defense pathways may function antagonistically or synergistically, depending on the timing, level and interaction between the host plant and aphid species (Pieterse et al., 2009). Therefore, in our study, this antagonism and synergism may be the reason that the inhibition of JA-responsive gene expression and JA accumulation by *H. defensa* infection was less pronounced and slower than that of SA-responsive gene expression and accumulation. Plant attack by herbivores is associated with oxidative damage at the cellular level through the accumulation of reactive oxygen species (ROS) (Kariola et al., 2005). Several protective enzymes, such as POD and PPO, are involved in ROS detoxification. Therefore, enhancement of the activities of these protective enzymes is one of the most essential elements of plant defense responses (Bednarski et al., 2013). Moreover, the induction of plant defense responses, such as the SA/JA defense pathway, can be influenced by ROS accumulation (Baker et al., 1997). In our study, the activity of the defense-related enzymes PPO and POD in plants was decreased after feeding by the *Hamiltonella*-infected clone, indicating that *H. defensa* can help aphids overcome the host plant defense response in some ways. The result was similar to that of a previous study in photo beetle larvae (Chung et al., 2013).

## Conclusion

Insect symbionts are newly identified participants that mediate plant-insect interactions during their long-term coevolution and play important roles in the defense and antidefense responses of plants and insects, respectively. Although we did not investigate in this study whether this defense suppression was induced by *H. defensa* or proteins and other molecules originating from *H. defensa* invasion in aphid oral secretions, our previous work has indicated that *H. defensa* can be detected in the aphid stylet and horizontally transmitted into wheat plants (Qian et al., 2018). Therefore, in combination with our previous work, this study has revealed antidefense strategies in aphids through *H. defensa* infection in response to the plant defense response. Notably, in our study, we verified the function of a specific *H. defensa* genotype in inhibiting plant defense responses using different *S. miscanthi* clones with identical genetic backgrounds, whereas in nature, aphids exist with additional strains of *H. defensa* (Chevignon et al., 2018) or with different genetic backgrounds. Thus, future studies are required to establish whether this difference is a general effect or a specific attribute of this particular combination of aphid and symbiont genotypes. Symbionts exist in many pests of important crops; thus, understanding the symbiont-mediated mechanism responsible for the suppression of plant defenses may provide important guidance to field arrangements of wheat varieties and the application of reasonable insecticide doses to reduce the potential threat of aphid outbreaks caused by symbiont-mediated aphid fitness enhancement. In addition, these results help generate a strategy for meeting the increased demand for novel insect pest management created by a growing human population and global climate change.

## Data Availability Statement

The raw data supporting the conclusions of this manuscript will be made available by the authors, without undue reservation, to any qualified researcher.

## Author Contributions

QL, JF, JC, and MH conceived and designed the experiments. QL, JS, and YZ performed the experiments. QL analyzed the data. QL and JC wrote the manuscript. All the authors read and approved the final version of the manuscript.

## Conflict of Interest

The authors declare that the research was conducted in the absence of any commercial or financial relationships that could be construed as a potential conflict of interest.
