# Supplementary material for: Anti-plant Defense Response Strategies Mediated by the Secondary Symbiont Hamiltonella defensa in the Wheat Aphid Sitobion miscanthi
Source: Front Microbiol. 2019 Oct 25;10:2419. doi: 10.3389/fmicb.2019.02419 (PMC6823553; doi:10.3389/fmicb.2019.02419)
Supplement: Supplementary file 1 [file Data_Sheet_1.pdf]

Supplementary Material

1 SUPPLEMENTARY METHODS

1.1 Details about microinjection and antibiotic treatment

Microinjection method

We collected 0.5 µl of hemolymph of the native *Hamiltonella*-infected *S. miscanthi* YX clone was collected and diluted it with 0.5 µl 0.01 M PBS solution, and then a 0.1 µl of the dilution was injected into the body of third-instar nymphs of the *Hamiltonella*-free DZ clone to build a *Hamiltonella*-infected *S. miscanthi* clone (called DZ-H) with an identical genetic background to that of the DZ clone. Following injection, recipient aphids were maintained in petri dishes containing healthy wheat leaves. Each artificially infected clone was derived from a single injected individual and screened regularly to confirm the presence of *H. defensa*.

Antibiotic treatment

Briefly, 30 3rd-instar aphids from the DZ-H clone fed on an artificial diet mixed with an antibiotic cocktail of 100 µg/ml each of ampicillin, cefotaxime and gentamycin for 3 days. The feeding apparatus consisted of 400 µl of the solution diet sandwiched between two layers of parafilm membrane and stretched around a glass tube with a 21-mm diameter under sterile conditions. The aphids that fed on the artificial antibiotic solution are referred to as *Hamiltonella*-cured aphids.

To rule out the effect of other secondary symbionts (*R. insecticola* and *Spiroplasma*) on follow-up experiments, an additional antibiotic treatment was performed. As before, 10 3rd-instar aphids from the DZ, DZ-H and DZ-HT clone fed on an artificial diet mixed with an antibiotic cocktail of 50 µg/ml each of ampicillin and tetracycline for 2 days to eliminate *R. insecticola* and *Spiroplasma* according to the previous study (Tsuchida et al., 2004; Simon et al., 2007; Jiggins et al., 2000). The surviving treated aphids were individually reared on wheat seedlings until reaching the adult stage, and their nymphs deposited from 24 to 48h after antibiotic treatment were collected. These nymphs were defined as G1 of each of the isofemale lines. From the G1 nymphs, ten aphids were randomly selected and reared on the plant until they reached the adult stage and produced a sufficient number of G2 offspring. The G1 mothers were then subjected to specific PCR detection to confirm the elimination of *R. insecticola* and *Spiroplasma*, and ten G2 nymphs were randomly selected and reared under the same conditions to obtain G3 offspring. In this way, several *R. insecticola* and *Spiroplasma* eliminated aphid clones that had been diagnosed as *Regella-Spiroplasma*-free in both of the G2 and G3 generations were established. Following antibiotic treatment, all of the surviving aphids were individually placed on wheat seedlings, the offspring were collected, and infection absence was confirmed using PCR for *H. defensa*, *R. insecticola* and *Spiroplasma*.

1.2 Details about fluorescence in situ hybridization (FISH)

Aphid embryos were dissected from adult aphids of the *Hamiltonella*-infected clone DZ-H and fixed for approximately 12 h in Carnoy's solution (chloroform-ethanol-acetic acid [6:3:1]). The samples were decolorized with 6% H_2O_2 in ethanol for 8 h and subjected to whole-mount FISH.

The embryos were incubated in prehybridization buffer (20 mM Tris-HCl [pH = 8.0]), 0.9 M NaCl, 0.01% sodium dodecyl sulfate, 30% formamide) without probe three times at 1 h each, and the samples were then incubated overnight in hybridization buffer containing 100 pmol of fluorescent probes per milliliter. Next, the samples were thoroughly washed in washing buffer (0.3 M NaCl, 0.03 M sodium citrate, 0.01% sodium dodecyl sulfate) three times for a half an hour each, mounted in Slow-Fade antifade solution, and then observed under a laser confocal microscope (LSM T-PMT 880, Carl Zeiss, NY).

2 SUPPLEMENTARY TABLES AND FIGURES

2.1 Figure

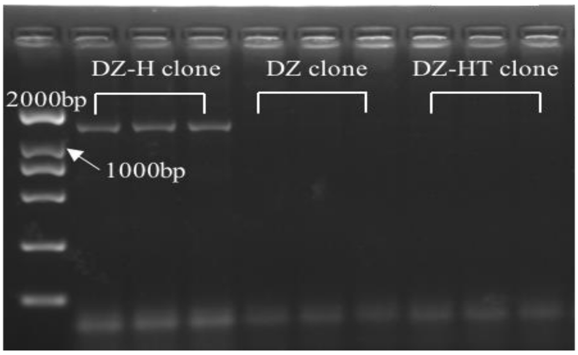

Figure S1. PCR detection of *H. defensa* in different aphid clones after microinjection and antibiotic treatment DZ-H clone: *Hamitonella*-infected clone by microinjection; DZ clone: native *Hamiltonella*-free clone; DZ-HT clone: *Hamiltonella*-cured clone by antibiotic treatment.

2.2 Table

Table S1: Primers used in this study

Application	Target	Primer name	Sequence (5'-3')	References
PCR	*COI*	COI-F	ACCAGTTTTAGCAGGTGCTATTAC	Favret et al., 2004
		COI-R	GTATATCGAGGTATACCATTT	
	dnaA	dnaA-F	GTTTTGTTTTGGATTGGGTTC	Henry et al., 2013
		dnaA-R	TCAGGAAGACTATGATTGGTGAG	
	recJ	recJ-F	ATCCGCTCTCAGAAACATACC	Henry et al., 2013
		recJ-R	GATGACATAAATCCAATGCCTC	
	P3	P3-F	TCGGGCGTAGTGTTAATGAC	Henry et al., 2013
		P3-R	TTCCATAGCGGAATCAAAGG	
qPCR (wheat)	*PR-1*	*PR-1*-F	ATAACCTCGGCGTCTTCAT	In this study
		PR-1-R	TACTCGCTCGGTCCCTCT	
	PAL	*PAL*-F	GGTCTTGTCTGCGGTGTTCT	In this study
		PAL-R	TTCGGCTTCATCAAGGGGTC	
	ß-1,3-GA	*ß-1,3-GA*-F	GAGGAGCTTCGGGGCTCTTCA	In this study
		ß-1,3-GA-R	ACGTGCCCGTTACACTTGGA	
	AOS	*AOS*-F	ACTTCAACACGCTCAACGACT	In this study
		AOS-R	TCACCGCTGACAAAGATGG	
	LOX	*LOX*-F	GACCAGCGAAACAACAACC	In this study

	LOX-R	GCATACAATAGCGGGAACAC	
FAD	*FAD*-F	TCCCATTCCACCTACTGC	In this study
	FAD-R	GGACTCACCAATCCGAGA	
ß-actin	*ß-actin*-F	GGAAAATCAGTCTCGGTTCAG	In this study
	ß-actin-R	TCATACAGCAGGCAAGCAC	

REFERENCES

1. Tsuchida, T., Koga, R., Fukatsu, T. (2004). Host plant specialization governed by facultative symbiont. Science. 303, 1989.
2. Simon, J. C., Sakurai, M., Bonhomme, J., Suchida, T., Koga, R., & Fukatsu, T. (2007). Elimination of a specialised facultative symbiont does not affect the reproductive mode of its aphid host. Ecol. Entomol. 32, 296-301.
3. Jiggins, F. M., Hurst, G.D.D., Dolman, C.E., & Majerus, M.E.N. (2000). High-prevalence male-killing *Wolbachia* in the butterfly *Acraea encedana*. J. Evol. Biol. 13, 495-501.
4. Favret, C., & Voegtlin, D.J. (2004). Speciation by host-switching in *Pinyon cinara* (insecta: Hemiptera: Aphididae). Mol. Phylog. Evol. 32, 139-151.
5. Henry, L.M., Peccoud, J., Simon, J.C., Hadfield, J.D., Maiden, M.J.C., Ferrari, J., Godfray, H.C. (2013). Horizontally Transmitted Symbionts and Host Colonization of Ecological Niches. Curr. Biol. 23, 1713-1717.
